# Potassium Channels, Glucose Metabolism and Glycosylation in Cancer Cells

**DOI:** 10.3390/ijms24097942

**Published:** 2023-04-27

**Authors:** Agata Wawrzkiewicz-Jałowiecka, Anna Lalik, Agnieszka Lukasiak, Monika Richter-Laskowska, Paulina Trybek, Maciej Ejfler, Maciej Opałka, Sonia Wardejn, Domenico V. Delfino

**Affiliations:** 1 Department of Physical Chemistry and Technology of Polymers, Silesian University of Technology, 44-100 Gliwice, Poland; 2 Department of Systems Biology and Engineering, Silesian University of Technology, 44-100 Gliwice, Poland; 3 Biotechnology Center, Silesian University of Technology, 44-100 Gliwice, Poland; 4 Department of Physics and Biophysics, Institute of Biology, Warsaw University of Life Sciences, 02-776 Warsaw, Poland; 5 The Centre for Biomedical Engineering, Łukasiewicz Research Network—Krakow Institute of Technology, 30-418 Krakow, Poland; 6 Institute of Physics, University of Silesia in Katowice, 41-500 Chorzów, Poland; 7 Faculty of Automatic Control, Electronics and Computer Science, Silesian University of Technology, 44-100 Gliwice, Poland; 8 Section of Pharmacology, Department of Medicine and Surgery, University of Perugia, 06129 Perugia, Italy

**Keywords:** potassium channels, cancer metabolism, Warburg effect, glycolysis, glutaminolysis, TCA cycle, glycosylation, channel glycanes, microRNA, cancer biomarkers

## Abstract

Potassium channels emerge as one of the crucial groups of proteins that shape the biology of cancer cells. Their involvement in processes like cell growth, migration, or electric signaling, seems obvious. However, the relationship between the function of $K^{+}$ channels, glucose metabolism, and cancer glycome appears much more intriguing. Among the typical hallmarks of cancer, one can mention the switch to aerobic glycolysis as the most favorable mechanism for glucose metabolism and glycome alterations. This review outlines the interconnections between the expression and activity of potassium channels, carbohydrate metabolism, and altered glycosylation in cancer cells, which have not been broadly discussed in the literature hitherto. Moreover, we propose the potential mediators for the described relations (e.g., enzymes, microRNAs) and the novel promising directions (e.g., glycans-orinented drugs) for further research.

## 1. Introduction

Throughout the years, advances in experimental techniques used in cellular and molecular have biology allowed the scientific community to make significant progress in our understanding of cancer pathogenesis and progression, which is gathered by the gradual improvement of therapeutic methods including, e.g., chemotherapy, radiotherapy, and immunotherapy. Still, however, the high incidence (over 19 million new cases per year), severity, and mortality of oncological diseases (ca. 10 million per year) remain one of the biggest global healthcare problems [1], which stimulates further search for new promising anti-cancer drug targets.

In that context, ion channels are gaining international attention due to their ability to regulate many aspects of cancer cells’ biology [2,3]. The ion channels enable the rapid and selective transport of ions through the biological membranes. Thus, its activity is critical for the fundamental tumor cell functions ranging from electrical excitation to cellular motility, which include cell volume regulation, migration, cell cycle progression, proliferation and apoptosis [2]. Thus, ion channels are one of the key proteins responsible for tumor cell survival and metastasis. It turns out that the altered expression of ion channels can be considered as one of the hallmarks of cancer, and several ion channel types have been linked to cancer cell chemoresistance [4]. From this perspective, ion channels emerge as a promising group of proteins that can be targeted in cancer treatment [5,6,7,8,9,10,11,12]. Not only the channels from the plasma membrane are involved in important physiological processes in cancer cells but also the ones present in its organelles, where mitochondrial channels are assumed to have a significant role as regulators of bioenergetics (ATP synthesis), intracellular $Ca^{2+}$ homeostasis, production of reactive oxygen species, and apoptosis [3,13]. In this work, we will discuss the relatively rarely considered aspects of the ion channel activity in cancer cells—namely, their roles in carbohydrate metabolism and the consequences of altered glycosylation of channels for cancer biology. We restrict ourselves to the broadest ion channel family, i.e., potassium channels. Potassium channels are transmembrane proteins responsible for the fast and selective transport of K${}^{+}$ ions through biological membranes down the electrochemical gradient. They are categorized into four main classes: calcium-activated potassium channels (K${}_{Ca}$), voltage-gated potassium channels (Kv), inward-rectifying potassium channels (Kir), and two-pore domain potassium channels (K${}_{2P}$) [7].

Carbohydrates are the main, but not the only, source of energy for the human body (in their digested state, when they are converted into glucose). They can also be important components of the molecular structure of different constituents of the cells in the form of glycans, which are formed in a process called glycosylation. It turns out that cancer cells are characterized by both heterogeneous changes in carbohydrate metabolism and glycome alterations [14,15].

Among the most typical metabolic changes occurring in cancer cells, one should mention: enhanced glucose consumption, increased glycolysis associated with reduced pyruvate oxidation, and a higher rate of lactic acid production (glutaminolysis), which are called the ”Warburg effect”. The complete mechanism of metabolic reprogramming from the preferential energy source of oxidative phosphorylation (OxPhos) in the mitochondria to glycolysis and glutaminolysis (fermentation) is yet to be explained. The main consequences of this phenomenon are disturbances in mitochondrial function [16,17], including, among others, the broadly observed changes in the expression of crucial enzymes involved in metabolism regulation [18,19,20,21], which lead to extracellular acidification, and eventually accelerate malignant progression.

Among the plethora of intriguing aspects of cancer biology, the formation of phenotype-specific glycoconjugates should also be highlighted. It turns out that tumor cells exhibit some innate characteristics of the glycans formed by their proteins and lipids, which can drive metastatic properties, inhibit apoptosis, or yield resistance to chemotherapy [15,22,23].

The compelling question one can ask is: are there any interconnections between impaired glucose metabolism and glycosylation in cancer cells? It turns out that there are a plethora of examples where both processes can influence each other. First, the increase in glucose and glutamine metabolism in cancer cells provides nutrient transporters and specific enzymes required in downstream anabolic pathways, including the biosynthesis of proteins, lipids, nucleic acids, and glycoconjugates. In particular, fructose 6-phosphate, glutamine, and acetyl coenzyme A (acetyl-CoA) are critical metabolites and also substrates of the hexosamine biosynthesis pathway to UDP-N-acetylglucosamine (UDP-GlcNAc). In turn, the UDP-GlcNAc concentrations are essential for the glycosylation processes leading to the biosynthesis of glycosaminoglycan, O- and N-linked glycans, as well as O-linked-N-acetylglucosaminylation (O-GlcNAcylation) [24,25,26].

As can be observed, metabolism and glycosylation are mutually associated, and both exhibit aberrations in cancer cells, which results in shaping the characteristic microenvironment that facilitates proliferation, distinctive signaling, angiogenesis, and metastasis. This cancer microenvironment is, however, influenced by many factors, including dysregulation of $K^{+}$ homeostasis by abnormalities in the expression and activity of potassium channels. In turn, the intra- and extracellular $K^{+}$ concentrations are one of the regulating factors for glycolysis and OxPhos [13,27]. Considering the altered glycome in cancer, it affects—globally—the biophysical properties of membranes [28] (thus, it indirectly influences the potassium channels’ activity via protein–lipid and protein–protein interactions [29,30]) and—locally—the glycosylation of the structure of the channels, which predefines their functionality [31,32,33,34,35]. In this work, we focus on the second aspect, which more evidently affects the channel activity. As one can infer, there is a complex biochemical metabolic network that interconnects the $K^{+}$ channels functioning, preferred pathways of glucose metabolism, and carbohydrate-based modifications of the structural components of cancer cells at a molecular level, which inspired us to synthesize the current state of knowledge in this subject. This overview outlines the relations between potassium channels, metabolic switches, and the consequences of the changes in glycans’ composition for the biology of cancer cells. Particular attention is paid to the possible molecular mechanisms and mediating molecules (e.g., non-coding RNAs or enzymes). This strategy allows us to propose promising directions for further in-depth research in this area.

## 2. The Role of Potassium Channels in the Metabolism of Cancer Cells

**Figure 1:**
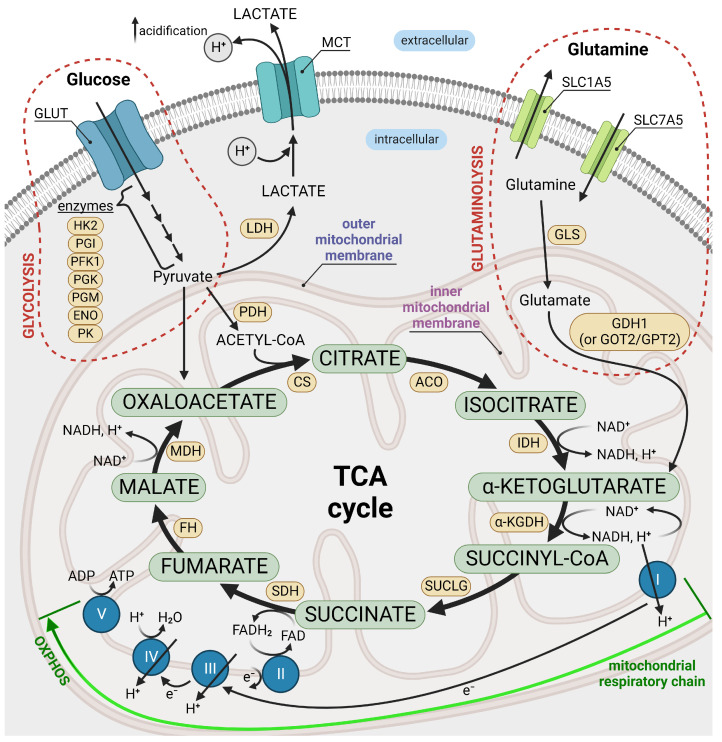
A simplified picture of cancer metabolism. Glycolysis is a multistage process of glucose transformation into pyruvate in the presence of specific enzymes. The obtained pyruvate can be further converted to lactic acid (aerobic glycolysis) or access the tricarboxylic acid (TCA) cycle, which leads to its full metabolization to CO${}_{2}$. The NADH and FADH${}_{2}$ released during the TCA cycle trigger the electron transport chain, which gives rise to oxidative phosphorylation (OxPhos). It turns out that aerobic glycolysis is a prime metabolic pathway to provide energy in cancer cells, while pyruvate oxidation is reduced, despite its higher energetic efficiency. Due to the high demand for NADH and the TCA cycle intermediates (needed to synthesize amino acids, nucleotides, and lipids), cancer cells render glutamine to sustain the tricarboxylic acid cycle in a process called glutaminolysis, where glutamine is converted to glutamate and further to α-ketoglutarate in the presence of glutaminase (GLS), GDH1, and/or other enzymes to, finally, enter the TCA cycle. Created with BioRender.com, (accessed on 27 September 2022).

The metabolism of cancer cells is shaped by a variable proportion of oxidative phosphorylation, glycolysis, and glutaminolysis (Figure 1), which depends on the tumor type [36]. Among the typical metabolic features of cancer cells, one can list higher consumption of glucose compared with their non-transformed couplings, enhanced aerobic conversion of pyruvate to lactic acid (“Warburg effect”), and, consequently, excessive production of acidic metabolic products [37,38].

In the following Sections we describe the relationships between the potassium channels’ functioning and the crucial component processes of cancer metabolism. The particular interest is paid on the glycolysis as a preferred source of energy, enhanced glutaminolysis, hampered tricarboxylic acid cycles, and the effects of hypoxia in cancer cells.

### 2.1. Glycolysis

Glycolysis is considered the primary process of cellular respiration. Simply, it involves breaking glucose into two three-carbon molecules called pyruvate. In general, it is a multistage process which can be summarized as follows:

(1) $$\mathrm{Glucose}\;+\;2{\mathrm{NAD}}^{+}+2\mathrm{ADP}\;+\;2P_{i}\to 2\mathrm{Pyruvate}\;+\;2\mathrm{NADH}\;+\;2\mathrm{ATP}\;+\;2H_{2}0\;+\;2H^{+}.$$

This process yields two ATP molecules containing free energy, two pyruvate molecules, two high-energy electron-carrying molecules of NADH, and two water molecules. In normal circumstances, generated pyruvate is used in the subsequent stages of cellular respiration to produce more ATPS. These processes (pyruvate oxidation, TCA cycle, oxidative phosphorylation) require, however, the presence of oxygen. Under hypoxic conditions, the pyruvate transforms into lactate, during which NADH drops its electrons off, turning into NAD${}^{+}$. It ensures a constant supply of NAD${}^{+}$ needed for glycolysis to keep running.

**Figure 2:**
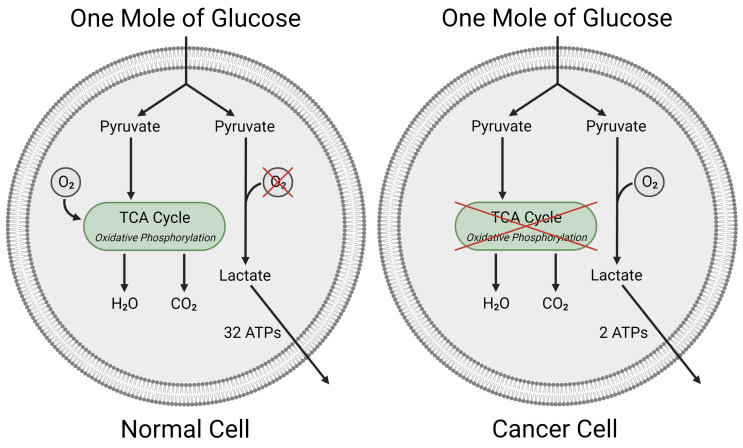
The simplified process of glycolysis occurring under hypoxic conditions in normal (**left** panel) cells and at normoxic conditions in cancer cells (**right** panel). In normal cells, the glucose is transformed into pyruvate which in the presence of oxygen participates in extremely energetic process called *oxidative phosphorylation* returning in total 32 ATPs. When the oxygen supplies are limited, the pyruvates turns into lactate resulting only in 2 ATPS. In tumour cells lactate is produced from pyruvate under hypoxic conditions. To counterbalance the deficiency in ATPs cancer cells have a 10- to 40-fold higher glucose uptake rate, and a lactate production which is 10–100 times faster than the complete oxidation of glucose in mitochondria (‘facilitated glycolytic flux’) in order to maintain energy homeostasis. Lactate is accumulated in the tumor extracellular space upon export from cancer cells (**right** panel). Created with BioRender.com, (accessed on 27 September 2022).

In cancer cells, the process of glycolysis is similar to that occurring in normal cells under hypoxic conditions (as presented in Figure 2 (left panel)). A hypoxic cellular environment is present in some tumors. Nevertheless, glycolysis may also be predominant in cancer cells even under normoxic conditions (Figure 2 (right panel)). Simultaneously, the consumption of glucose is higher in comparison to normal cells. It results in a higher rate of glycolysis associated with reduced pyruvate oxidation and increased production of lactate. This “injured” form of respiration is called *the Warburg effect* [39,40] and is considered one of the hallmarks of cancer cells. This persistent aerobic glycolysis can be associated with the activation of oncogenes or the loss of tumor suppressors in certain neoplastic cells [41,42,43,44,45]. The Warburg effect alone does not explain why cancer cells abandon mitochondrial respiration in favor of much less efficient aerobic glycolysis. For comparison, from the mitochondrial oxidative phosphorylation we obtain 36 ATP vs. only 2 ATP generated from glycolysis. It seems, however, that glycolysis is faster and may be more suitable for tumor cells’ vital functions maintenance as long as constant glucose supplies are ensured [44,46]. It may also be advantageous in the process of adaptation to hypoxic conditions in the early phase of tumor development, where access to oxygen is limited. Moreover, glycolytic energy metabolism of tumor cells is beneficial for perpetual proliferation and meeting the high demand for non-essential amino acids, fatty acids, and nucleotides [47]. Alternatively, adjustment to an acidic microenvironment occurring in the tumor cells due to excessive lactate production may also promote the evolution of the glycolytic phenotype [48,49].

Warburg initially explained this impaired form of metabolism as mitochondrial dysfunctionality [50]. Recent findings indicate, however, that the increased rate of ATP production by glycolysis occurs in cells in which mitochondria are not damaged [16,42]. Nowadays, the Warburg effect is considered a consequence of mutations in mitochondrial enzymes, such as fumarate hydratase, succinate dehydrogenase, and isocitrate dehydrogenase, and by excessive production of mitochondrial reactive oxygen species (ROS) [14,51,52,53,54]. It is also regarded as the interplay between the normoxic/hypoxic activation of the transcription factor hypoxia-inducible factor-1 (HIF-1), oncogene activation, loss of function of tumor suppressors, altered signaling pathways and interaction with components of the tumor microenvironment (TME) [17].

**Figure 3:**
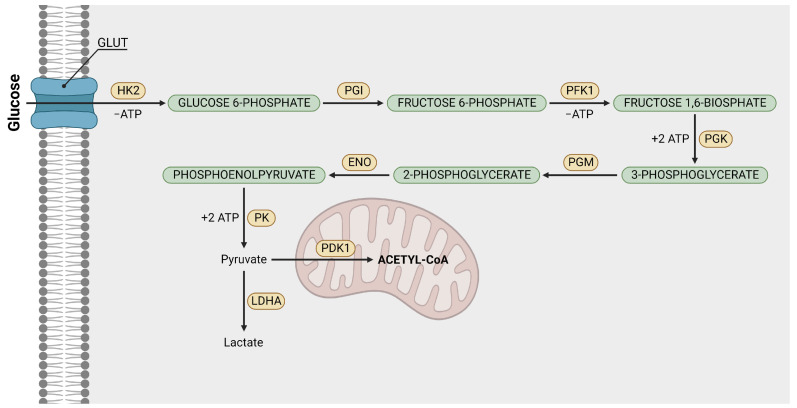
Key biochemical steps of the aerobic glycolysis. Glucose is imported into cell through overexpressed GLUT transporters. It is then transformed into pyruvate through 9–10 chemical reactions accelerated by different enzymes placed in green boxes. They are hexokinase-II (HK2), phosphoglucose isomerase (PGI), phosphofructokinase (PFK), phosphoglycerate kinase (PGK), phosphoglycerate mutase (PGM), enolase (Eno), pyruvate kinase (PK) and lactate dehydrogenase A (LDHA). At the end of the process, pyruvate is converted into lactate which is transported outside the cell with upregulated monocarboxylate transporter 4 (MCT4). The pyruvate dehydrogenase kinase 1 (PDK1) inhibits the intra-mitochondrial conversion of pyruvate to acetyl-CoA. Note that the whole process produces only 2 ATPs in total (2 ATPs are consumed in order to return 4 ATPs). Created with BioRender.com, (accessed on 27 September 2022).

In Figure 2, we present a very simplified scheme of aerobic glycolysis as a part of the metabolic pathway in neoplastic cells. In reality, the catabolism of glucose to pyruvate is a complicated process involving 9–10 biochemical steps. The most important ones are presented in Figure 3. The glucose is imported into cell with upregulated GLUT transporters [47]. Then, it is transformed into pyruvate through biochemical reactions catalyzed by enzymes: hexokinases, phosphoglucose isomerase (PGI), phosphofructokinase I (PFK1), phosphoglycerate kinase (PGK), phosphoglycerate mutase (PGM), enolase, pyruvate kinase (PK) and lactate dehydrogenase A (LDHA). Afterwards, pyruvate is transformed into lactate, which is subsequently ejected from the cell with pregulated monocarboxylate transporter 4 (MCT4). Then, it is accumulated in the extracellular space. Simultaneously, mitochondrial pyruvate dehydrogenase kinase 1 (PDK1) impedes the conversion of pyruvate to acetyl-CoA [17].

Having the above-described process in mind, we ask the following question: are we able to prevent tumor growth by forcing cells to produce energy with aerobic metabolism [55,56]? To answer this query, we need to better understand the glycolytic energy metabolism and action mechanism of most crucial enzymes, which become upregulated in most neoplastic cells [57]. One of the possible directions toward the development of novel anticancer strategies is to study the impact of potassium ion channels on aerobic glycolysis. In general, it has been reported that the presence or absence of these channels may affect the activity of enzymes taking part in glycolysis, oxidative phosphorylation, and the tricarboxylic acid (TCA) cycle [58,59]. It, in turn, is associated with an alternating cancer malignancy and influences the rate of cell division, migration, metastasis, migration, and resistance to anti-cancer therapies.

As for glycolysis, one of the most important enzymes catalyzing the process is pyruvate kinase (PK). Its main task consists of the conversion of phosphoenolpyruvate and ADP to pyruvate and ATP (Figure 3) in the presence of ions such as $K^{+}$ or $Mg^{2+}$. Therefore, it is likely that abnormal $K^{+}$ channel expression affects this reaction [60]. Another $K^{+}$–dependent step in aerobic glycolysis is the transformation of glucose into glucose-6-phosphate which is catalyzed by hexokinase II. This enzyme is considered a crucial metabolic switch toward aerobic glycolysis. It has been demonstrated in refs. [27,61] that a decrease in intracellular $K^{+}$ ions impaired aerobic glycolysis and triggered energy stress pathways. The re-addition of the potassium ions could restore the ATPs generation via glycolysis. In ref. [62] it was underlined that the interaction of overexpressed hexokinase I (HKI) with VDAC (*Voltage-Dependent Anion Channel*) supports GLT-1 (*glutamate transporter*)-mediated transport activity. Another study confirmed the impact of the K${}_{2P}$3.1 ion channel (considered a representative of the two-pore domain $K^{+}$ channels) on glucose metabolism in lung cancer cells [63]. Results demonstrated that overexpression of K${}_{2P}$3.1 was responsible for diminished glucose uptake and, as a consequence, for a decreased rate of lactate production. Furthermore, it was shown that elevated expression of these potassium channels can lead to the downregulation of GLUT1 and LDHA.

It is also worth mentioning the review [64], which gathers knowledge about relationship between the Warburg effect and PAH (*Pulmonary Artery Hypertension*). It turns out that, similarly to cancer cells, the metabolism in pulmonary artery cells is altered; the ATPs are obtained through aerobic glycolysis rather than from mitochondrial oxidative phosphorylation. There are several therapeutic strategies that inhibit the development of this disease. One of them involves DCA Dichloroacetate which is an analog of acetic acid the inhibits the action of PDK1 (Figure 3) and enhances oxydative phosphorylation [65]. As previously emphasized, this enzyme impedes the conversion of pyruvate into acetyl-CoA. Nonetheless, DCA can stop this process [65,66,67] in the pulmonary artery as well as in cancer cells through the regulation of potassium channels. It has been demonstrated that, on contrary to normal cells, neoplastic cells are characterized by a high DJm (*mitochondrial membrane potential*) and low expression of the voltage–dependent potassium channel Kv1.5. Both these factors contribute to apoptosis resistance. Besides impeding the PDK1 (which results in metabolism alteration towards glucose oxidation) DCA decreases DJm, upregulates mitochondrial $H_{2}O_{2}$ (relatively stable ROS *reactive oxygen species*), and stimulates the activity of potassium ion channels [13].

Yet in another study, the impact of small-conductance calcium-activated $K^{+}$ (SK) channels was investigated [68]. The authors revealed that CyPPA, an activator of SK channels, slightly reduced the level of mitochondrial respiration in favor of glycolysis and lactate production. In agreement with the studies, it was shown that DCA can reverse the process.

It has also been reported that astrocytes in vitro and in vivo maintain a cytosolic reservoir of lactate, which upregulates the plasma membrane $K^{+}$ ion channels. It results in the fast transport of $K^{+}$ ions to the extracellular space [69].

In ref. [70], it was pointed out that aerobic glycolysis in cancer cells generates an abundance of protons in a gradient across most solid tumors with an acidic core and an alkaline rim. The authors discovered that the rate of cell proliferation depends on extracellular pH. In the example of glioma cells, they demonstrated that changes in pH are detected by $H^{+}$–sensitive $K^{+}$ ion channels, which translate the changes in pH into changes in membrane potential. These tonically active potassium channels can be blocked by quinine and ruthenium red. The downregulation of $K^{+}$ channels leads to glioma cells depolarization and, consequently, stops their proliferation.

The article [71] focused on the role of the K${}_{Ca}$3.1 channel in Liver Cancer Stem Cells (LCSC). The studies revealed enhanced expression of these potassium channels in LCSCs. The elevated K${}_{Ca}$3.1 led, in turn, to the upregulation of enzymes catalyzing the reactions of glycolysis, such as HK2 or PFK1.

In ref. [72], it was demonstrated that there exists a correlation between the expression of GLUT transporter and the expression of Kv11.1 (hERG) channels in colorectal cancer cells. The presence of hERG turned out to be associated with a lack of GLUT expression and resulted in a worse prognosis for patients suffering from this kind of neoplasm.

The authors of [73] provided direct evidence for interaction between Kir6.2 subunits in rat ventricles and pyruvate kinase. Since the expression of pyruvate kinase is increased in most tumor cells, this interaction may lead to their alternated metabolism.

In search of the potential modulators that shape the relations between ion channels and metabolic pathways in cancer, we paid particular attention to microRNAs. In recent work, Mirzaei and Hamblin cataloged microRNAs, which are associated with the cancer-related changes in glycolytic pathways according to experimental in vitro and/or in vivo models. To find a possible relation between the K${}^{+}$ channels and cancer metabolism, we searched for the common microRNAs from the ones presented in [74] and the ones that are anticipated to regulate the expression of potassium channels according to at least two from three independent databases TargetScan [75], MirTarBase [76,77], and MirDB [78]. The results are presented in Table 1.

As one can see in Table 1, the brief investigations allow us to conclude that there are microRNAs, which can simultaneously affect the crucial biomolecules responsible for the metabolic characteristics of cancer (like, e.g., HK1, HK2, LDHA) and the expression of some types of potassium channels. In that way, they can further indirectly modulate the electrical microenvironment of cancer to enhance its progression. What is more, most of the indicated microRNAs can be found in exosomes. Therefore, they can affect cellular pathways that significantly regulate cancer biology (tumor growth, invasion, metastasis, angiogenesis, and its resistance to drugs) [107].

### 2.2. Mitochondrial Link to Cancer Cell Metabolism of Glucose and Potassium Channels

#### 2.2.1. Mitochondrial Function

Mitochondria serve mainly as the powerhouse of the cell. However, mitochondrial function goes far beyond ATP production. They play prominent roles in the signaling pathways of many cellular processes. In cancer cells, mitochondria undergo many alterations, and their functioning is impaired, partly due to mutations encoding mitochondrial electron transport chain (ETC) complexes. Mitochondrial DNA mutations concerning complex I, III, and IV are well-described for various types of cancer [108]. Mitochondrial dysfunction in cancer cells also involves the impairment of key TCA cycle enzymes. Such enzymes as citrate synthase, aconitase, isocitrate dehydrogenase, succinate dehydrogenase, fumarate hydratase, and malic enzyme are upregulated. Others, such as oxoglutarate dehydrogenase, malate dehydrogenase, or pyruvate dehydrogenase, are downregulated [109]. Eventually, disruption of ETC as well as the TCA cycle leads to mitochondrial dysfunction. It can lead to aberrant mitochondrial ROS production and consequently regulate cancer cell metabolism. Increased ROS production is also correlated with the role of mitochondria in cell death regulation. This regulation involves apoptosis and cytochrome c release from mitochondria, as well as necroptosis and ROS-dependent MPTP activation [110].

#### 2.2.2. Glutaminolysis and the TCA Cycle

The transition processes of glutamine and glucose in rapidly dividing cancer cells are highly combined. Under the influence of glutaminase (GLS), the glutamine is transformed into glutamate, which is further converted to α-ketoglutarate. The latter takes part in energy production through the tricarboxylic acid cycle (the TCA cycle). However, an alternate scenario is also possible, where α-ketoglutarate produced from glutamate in the GDH reaction is used for reductive (counter-clockwise) metabolism in the TCA cycle. The reductive TCA cycle pathway yields isocitrate, citrate, and acetyl-CoA, which serve as substrates for lipogenesis and the synthesis of other biomass in rapidly dividing cancer cells [111,112,113]. These, in turn, support cell growth and viability [114,115,116].

It is well-known that glutaminolysis (when metabolized in the TCA cycle in the oxidative, clockwise, direction) is a valuable energy source in cancer cells. The key enzymes in the process of glutamine metabolism are strongly involved in tumorigenesis [117]. It is also proven that the TCA cycle mediates the inhibition or promotion of tumor progression [118]. For these reasons, glutaminolysis is frequently considered a possible target in cancer therapy [119]. The glutamine additionally participates in the synthesis of glutathione (GSH), which is also localized in mitochondria and has great importance in counteracting reactive oxygen species (ROS). It protects the cellular organelles from the action of ROS [120] and has a proven impact on cancer metabolism [121]. A higher level of ROS is found in almost all cancers [122]. Despite the fact that there is no direct relation between the glutaminolysis and the potassium channels activity, the activation of potassium channels may impact the GSH synthesis and final production of ROS.

#### 2.2.3. Hexokinase

**Figure 4:**
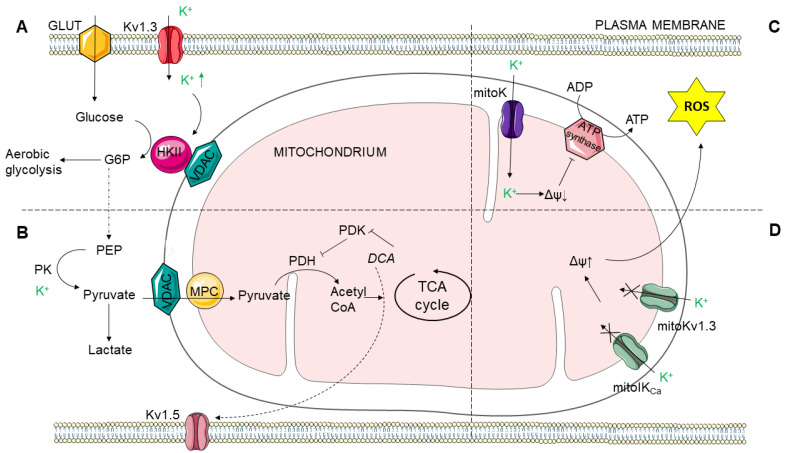
The relationship between K${}^{+}$ channels, metabolism of cancer cells, and mitochondria. (**A**) Glucose enters the cells via glucose transporters (GLUT). The glucose is transformed into glucose-6-phosphate (G6P) by hexokinase. Hexokinase II (HKII) is bound to the mitochondrial voltage-dependent anion channel (VDAC). HKII activity is regulated by the intracellular K${}^{+}$ ions and the plasma membrane-Kv1.3 channel activity. (**B**) G6P is transformed into phosphoenolpyruvate (PEP). Pyruvate kinase (PK) transforms PEP into pyruvate. PK requires K${}^{+}$ for its activity. Pyruvate can enter mitochondria via VDAC and mitochondrial pyruvate carrier (MPC) to fuel the TCA cycle. Pyruvate dehydrogenase (PDH) catalyzes the transformation of pyruvate to acetyl-CoA. PDH activity is controlled by K${}^{+}$-dependent pyruvate dehydrogenase kinase (PDK). Inhibition of PDK with dichloroacetate (DCA) is involved in the increased expression and activation of the Kv1.5 channel. (**C**) Mitochondrial potassium channels transport K${}^{+}$ across the inner mitochondrial membrane into the mitochondrial matrix. The K${}^{+}$ influx disrupts mitochondrial membrane potential (ΔΨ) generated by the electron transport chain, which affects ATP-synthase activity and reactive oxygen species (ROS) production. (**D**) Inhibition of voltage-dependent and calcium-activated mitochondrial potassium channels (mitoKv1.3 or mitoIK${}_{Ca}$) increases ΔΨ and subsequent ROS production in cancer cells. The Figure was partly generated using Servier Medical Art, provided by Servier, licensed under a Creative Commons Attribution 3.0 unported license.

Mitochondrial function is also strongly associated with the function of the crucial enzymes in glucose metabolism. The first critical enzyme in metabolism of glucose is hexokinase. Hexokinase is an enzyme that phosphorylates the glucose and forms glucose-6-phosphate. In humans, there are four hexokinase isoforms, where hexokinase I and II are related to mitochondria and are involved in cancer cell metabolism [123,124]. Hexokinase I and II are associated with the mitochondrial VDAC channel, and they are overexpressed in cancer cells. It was established that mitochondrial hexokinase is coupled with oxidative phosphorylation, and a high level of hexokinase correlates with high glycolytic activity in cancer cells [125]. Thus, hexokinase may serve as a switch between aerobic and anaerobic glycolysis. What is more, it was discovered that the activity of hexokinase II may be regulated by potassium ions and, thus, the channels in the plasma membrane (as presented in Figure 4A). Bischof and coworkers provided a link between Kv1.3 activity and cell metabolism. The increased expression and activation of the Kv1.3 channel leads to a decrease in intracellular K${}^{+}$ concentration, which in turn lowers the glycolytic activity of the cell [27]. The mechanism involves hexokinase II, which is highly expressed in cancer cells, and the K${}^{+}$ ions, which are essential to maintaining aerobic glycolysis activity.

#### 2.2.4. Pyruvate Kinase

Glucose-6-phosphate is then used to form pyruvate. The pyruvate can later enter either the TCA cycle to fuel oxidative phosphorylation in mitochondria in high oxygen conditions or be used to synthesize lactic acid in the cytoplasm in low oxygen conditions. The enzyme that catalyzes the conversion of phosphoenolpyruvate to the final product of glycolysis- pyruvate, is pyruvate kinase (PK). There are four isoforms of pyruvate kinases. Among them, PKM2 is highly expressed in tumor cells. PKM2 can form either a more active tetramer or a less active dimer. In cancer cells, PKM2 usually forms a less active dimer and promotes the conversion of pyruvate to lactic acid. Therefore, pyruvate kinase can act as a metabolic switch in cancer cells [126]. There is a potential role for potassium channels in the process, as the activity of PK requires K${}^{+}$, along with other monovalent cations [59] (Figure 4B).

#### 2.2.5. Pyruvate Dehydrogenase

Another enzyme that determines whether the metabolic pathway of glucose will lead to lactate production or to the oxidation pathway in mitochondria is pyruvate dehydrogenase (PDH). PDH is an enzyme that catalyzes the conversion of pyruvate to acetyl-CoA, which feeds the TCA cycle in mitochondria, producing electron donors such as NADH and FADH${}_{2}$. The activity of PDH is inhibited by PDH kinase (PDK), which phosphorylates PDH. What is interesting, the PDH and PDK activities are related to the functioning of the Kv1.5 potassium channels. It was discovered that inhibition of PDK with dichloroacetate (DCA) promotes glucose oxidation and activates Kv channels in cancer cells, but not in the normal ones [13]. Moreover, cancer cells show lower expression of the Kv1.5 channel, and DCA upregulates its expression in NFAT1-dependent way.

#### 2.2.6. Mitochondrial Potassium Channels

Potassium channels are present not only in the plasma membrane but also in various intracellular compartments and organelles, such as mitochondria. The potassium channels that were discovered in the inner mitochondrial membrane are ATP-sensitive (mitoK${}_{ATP}$), Ca${}^{2+}$-activated large-conductance (mitoBK${}_{Ca}$), Ca${}^{2+}$-activated intermediate-conductance (mitoIK${}_{Ca}$), Ca${}^{2+}$-activated small-conductance (mitoSK${}_{Ca}$), voltage-gated (mitoKv1.3 and mitoKv7.4), two-pore domain (mitoTASK-3), and sodium-activated (mitoSlo2) potassium channels [127]. According to the literature, mitoKv and mitoIK${}_{Ca}$ channels play substantial roles in cancer cells [9].

It is well-known that mitochondria play a pivotal role in cell death, while cancer cells escape this general route. Mitochondria-regulated cell death involves mitochondrial permeability transition pore (MPTP), which in turn is regulated by Ca${}^{2+}$ overload or by ROS overproduction [128,129]. Mitochondrial potassium channels are well known regulators of ROS production (Figure 4C,D). It was found that Kv1.3 and mitoKv1.3 channels are overexpressed in many tumor cells [8]. It was also discovered that inhibition of the mitoKv1.3 channel, either by Bax protein or Kv1.3 channel inhibitors, caused apoptosis in lymphocytes. The mechanism of this phenomenon involved K${}^{+}$-related hyperpolarization of the inner mitochondrial membrane, consequent ROS generation, MPTP opening, and cytochrome c release [130]. The studies on pancreatic ductal adenocarcinoma lines [131] confirm the role of mitoKv1.3 channels in the regulation of cancer cells’ apoptosis via modulation of the ROS-related signalling. The data concerning cancer cells also indicates that inhibition of mitoKv1.3 induces cell death, however, in a Bax-independent way [132]. Similarly to Kv1.3, K${}_{Ca}$3.1 channel is overexpressed in many cancer cells, such as malignant glioma or lung cancer [133,134]. This overexpression is related to promalignant properties of the channel. Moreover, the studies [135,136] confirmed the localisation of the K${}_{Ca}$ channels in the inner mitochondrial membrane in human colon and lung cancer cells. Likewise the Kv1.3 channels, inhibition of K${}_{Ca}$s (along with other anti-cancer drug administration) caused enhancement of apoptosis. The mechanism also involves changes in mitochondrial membrane potential and ROS overproduction [137]. The effect of potassium channels on mitochondria is associated not only with ROS production but also with the regulation of oxidative phosphorylation. In pancreatic ductal adenocarcinoma cells, it was discovered that K${}_{Ca}$3.1 channels regulate oxygen consumption, ATP production, and, consequently, cell proliferation. However, the studies do not clarify whether mitochondrial or plasma membrane channels are involved in this effect [138].

It is well known that mitochondrial potassium channels are involved in many cytoprotective effects. In the work of Malinska et al. [139], authors characterize the association between the mechanisms of the mitochondrial production of ROS and the potassium channels’ activation, which can result in cytoprotection. The activation of $K^{+}$ channels in the inner mitochondrial membrane entails a decrease in mitochondrial membrane potential and, consecutively, may lead to many different phenomena, including the deactivation of the mitochondrial intrinsic apoptosis pathway [140]. The specific mitoK${}_{ATP}$ and mitoBK${}_{Ca}$ channels may play a primary role in cytoprotection [141]. In the context of the glutamine to glutamate transformation, it is worth mentioning the role of the K${}_{Ca}$2.2 (SK2) channels in glutamate toxicity. The activation of these channels prevents mitochondrial super-oxide formation and reduces the risk of cellular apoptosis [142]. The glutamate-induced toxicity can also be reduced by the inhibition of the mitoBK${}_{Ca}$ channels using a popular BK${}_{Ca}$/mitoBK${}_{Ca}$ channel blocker, paxilline [143].

### 2.3. Effects of Hypoxia on the Potassium Channels Activity

Hypoxia is a common characteristic of tumors. It occurs when oxygen is not available in sufficient amounts to maintain proper homeostasis at the tissue level. Low availability of oxygen is a complex phenomenon triggered by uncontrolled cell proliferation, during which they quickly consume nutrients and oxygen from the vasculature [144], increased distance from blood vessels, and impaired blood flow caused by cell aggregates. Cancer cells, however, are able to adapt to the altered conditions through neo-angiogenesis, metabolic changes, and metastasis, which allows them to prolong proliferation. These adaptation paths, in turn, are associated with higher resistance of tumors to therapeutics applied and lead to higher aggressiveness of the tumor.

The main role in this process is played by hypoxia-inducible factor 1 (HIF-1). It is a dimeric protein complex regulating the cellular and homeostatic response to hypoxia [145]. It also drives aerobic glycolysis and prevents cells from hypoxia-stress damage [146]. Hypoxia leads to HIF-1 stabilization. Consequently, HIF-1 is translocated to the nucleus, where it takes part in the induction of transcription of multiple genes encoding transporters and enzymes necessary for cancer cells in the adaptation process. The HIF-1 level can be regulated by the potassium channels.

Hypoxia may alter the function of potassium channels either directly by affecting subunits of potassium channels or via various signaling molecules. Hypoxia induces depolarization of membrane potential by inhibiting the activity of several potassium channels. It was documented that hypoxia inhibits potassium channels such as voltage-gated (Kv1.2, Kv1.5, Kv2.1, Kv3.1, Kv3.3, Kv4.2, and Kv9.3) or two-pore domain potassium channel (TASK-1). In hypoxic conditions, these channels are closed, whereas in normoxic conditions, they remain open [147]. In cancer cells, it was shown that Kv3.1 and Kv3.4 are implicated in migration and invasion processes in a HIF-dependent way, involving the ERK (extra cellular signal-regulated kinase) pathway [148]. It is documented that the Kv10.1 channel is involved in the increased HIF-1 alpha expression and activity, which induces tumor angiogenesis via increased vascular endothelial growth factor (VEGF) [149]. Similarly, Kv11.1 is involved in tumor vascularization in colorectal cancer via VEGF secretion in a HIF-dependent way [150]. The action of HIF-1 has also been shown to correlate with the expression of Kv10.1 and Kv11.1 channels in the breast [151].

An intriguing relationship between potassium channels and hypoxia is observed not only for the effect of the channel proteins on second messengers in hypoxia but also for how hypoxia can affect the activity of the potassium channels. This complex interdependency was extensively reviewed recently by Girault et al. [152]. It has been found that hypoxia, either directly or via second messengers such as ROS, can alter the function of potassium channels. The effect on the BK${}_{Ca}$ channel varies depending on the localization of the channel protein. In plasma membrane, hypoxia inhibits BK${}_{Ca}$ channel. Whereas in mitochondria, it increases BK${}_{Ca}$ activity. The inhibitory effect of hypoxia on the potassium channels has also been documented for K${}_{2P}$3.1 (also known as TASK-1). Furthermore, the K${}_{2P}$3.1 channel is related to the changes in nutrient transport and the overexpression of Na${}^{+}$-coupled transporters for mio-inositol, biotin and glutamine, which is involved in metabolism changes [153]. In turn, the overexpressed two pore domain potassium channel KCNK9 gene (encoding K${}_{2P}$9.1) present in human breast and small-cell lung tumors can contribute to the resistance to hypoxic conditions [154]. A possible signalling mechanism linking hypoxia to ion channel function is alternation in ROS production. In neuroblastoma cells it has been shown that hypoxia reduces hERG (Kv11.1) channel by increasing ROS production. Increased ROS level interferes in the association of hERG with Hsp90 (Heat Shock Protein 90) and causes retention of the channel in endoplasmic reticulum [155]. In medulloblastoma the mechanism of Kv2.1 channel inhibition involves HO-1 (heme oxygenase-1; also known as Hsp32). HO-1 upregulation induces CO production and consequent Kv2.1 channel inhibition [156]). Lower activity of Kv1.5 channel in Erwing sarcoma and neuroblastoma cells can be associated with lower Kv1.5 channel expression [157] Another study shows that hydrogen sulphide may be implicated in hypoxia-induced radio resistance. The mechanism involved KATP channel activation by H_2_S [158].

## 3. Glycosylation-Dependent Alterations in Properties of Potassium Channels in Cancer Cells

Glycosylation, one of the most common post-translational modifications of proteins, is the enzymatic process of attaching mono- or oligosaccharides to protein molecules. Glycosylation occurs in the endoplasmic reticulum and in the Golgi apparatus. Unlike the processes of transcription and translation, glycosylation is a non-templated process [22]. Therefore, the final structure of the glycans attached to a given protein molecule is a product of the presence of specific glycosyltransferases and glycosidases in the immediate vicinity of the peptide, the availability of substrates, and the overall state of the cell. The most common types of glycosylation are N- and O-glycosylation (where glycans are attached to the protein chain by an N- and O-glycosidic bond, respectively). The asparagine, present in the sequence Asn-X-Thr/Ser (X any amino acid except proline), undergoes N-glycosylation. O-glycans are attached to a serine or threonine residue within the protein molecule (there is no consensus sequence). However, N-glycans have also been shown to be attached within a non-consensus sequence and O-glycans to side chains of amino acids other than Ser or Thr [159]. Oligosaccharides attached to proteins affect their structure, stability, and activity [160]. Sugar structures on the cell surface form a complex layer called the glycocalyx [161]. Glycans attached to macromolecules play crucial roles in cellular processes and intercellular interactions [162]. The alteration in the glycosylation pattern is closely related to physiological and pathological changes that occur in the cell [15].

### 3.1. Glycosylation in Cancer Cells

The glycosylation pattern of cancer cells differs significantly from that of normal cells [163]. Oligosaccharide structures overrepresented in cancer cells include branched high-mannose N-glycans, truncated O-glycans with a large number of O-GLCNAc (O-linked N-Acetyl-D-glucosamine), glycans with abnormal core fucosylation, and oligosaccharides containing terminal sialic acid [164]. The reasons for the changes in the glycosylation pattern are not yet fully understood. Previous studies suggest that the changes in glycosylation that occur in cancer cells may be related to the expression levels of enzymes involved in glycan processing, the relocation of these enzymes within the endoplasmic reticulum and the Golgi apparatus, and changes in the pH of the Golgi apparatus [165]. Glycan structures found in cancer cells are directly related to cancer development, progression, and metastasis [22]. Sialic acid has a negative charge, which mediates intercellular interactions and signal transduction [166]. Overexpression of sugar antigens (e.g., sialyl-Lewis A antigen) is associated with poor survival in cancer patients [166]. Inhibition of fucosylation leads to inhibition of the proliferation, migration, and invasion of cancer cells [22]. Studies with tunicamycin, an antibiotic that inhibits N-glycosylation, have shown that N-glycans promote tumor growth and metastasis [167].

### 3.2. Functional Role of Glycans in Potassium Channels

Potassium ion channels are protein tetramers that undergo numerous post-translational modifications. Each channel subunit contains transmembrane segments linked by linkers [168]. Research on the effects of oligosaccharides attached to potassium channels has focused mainly on N-glycosylation, although the K${}^{+}$ channels can also undergo O-glycosylation [169]. Most voltage-gated potassium Kv channels have one–two N-glycosylation sites located on the S1–S2 linker or two–three glycosylation sites in the S5-S6 linker [170,171]. Kv channels that lack an N-glycosylation site (e.g., Kv4.2, Kv4.3) are O-glycosylated [169]. Two-pore domain potassium channels (K${}_{2P}$) have one–two N-glycosylation sites, while no O-glycan was found [172]. Inward rectifier potassium channels (Kir) have one, not always occupied, N-glycosylation site [173].

Glycosylation of potassium channels affects their activity, dynamics, stability, and localization [170]. Studies in hamster cells have shown that sialic acid attached by an O-glycosidic bond modulates the gating of Kv4.2 and Kv4.3 potassium channels, but it does not affect their voltage-dependent steady-state inactivation [169]. In contrast, studies of the Kv12.2 channel, which has three N-glycosylation sites within the S5-S6 loop, showed that N-glycans regulate voltage-dependent activation of this channel in a sialic acid-independent manner. The same study also found that N-glycosylation of at least one (any) of the glycosylation sites is required for the expression of the Kv12.2 channel on the cell surface [171]. Similarly, the attachment of a sugar chain within the S1-S2 linker promotes the surface expression and stability of ion channels and directly affects their gating and voltage sensitivity [174]. Studies conducted on wild-type and mutant (lacking N-glycosylation sites) Kv3.1 channels suggest that glycosylation is essential for proper folding and expression of this channel on the cell surface and also affects the opening of the voltage-dependent gate of the Kv3.1 channel [175].

The role of glycans depends on the channel type, and even within the same subfamily, channels may respond differently. For example, studies have shown that the Kv1.5 channel gate is modulated by sialylated N-glycans, while no effect of desialylation/deglycosylation was observed on the Kv1.4 channel gating [176]. The authors suggest that the distinct effects of sialylation on the gating of the Kv1.5 and Kv1.4 channels may be due to the difference in length and amino acid composition of the S1–S2 linker.

The effect of oligosaccharides on potassium channels also depends on the composition and structure of the glycans themselves. Studies on the Kv3.1 channel proteins showed that the localization of these channels within the plasma membrane and their activity vary depending on the type (hybrid or complex) of the attached N-glycan [177]. The effect of glycosylation on the function and stability of Kv channels has been extensively studied, but in other types of potassium channels, attached N-glycans also play notable physiological roles. For example, deglycosylation has been shown to cause a decrease in the number of K${}_{2P}$3.1, K${}_{2P}$9.1, K${}_{2P}$2.1, and K${}_{2P}$17.1 channels in the cell membrane [33,172,178]. Furthermore, changes in the glycosylation of the K${}_{2P}$3.1, K${}_{2P}$2.1, and K${}_{2P}$17.1 channels have been linked to changes in membrane currents [33,172,178]. However, glycosylation does not affect the pH sensitivity of the K${}_{2P}$2.1 channel [178]. Glycosylation in Kir channels affects gating, the magnitude of macroscopic currents, and the number of channels on the cell surface [173,179].

### 3.3. miRNAs as a Common Element in the Regulation of Potassium Channels and Sialotransferases

The tumor microenvironment plays a pivotal role in cancer progression. One element in the communication of tumor cells with the surrounding microenvironment and with other cells is miRNAs (short non-coding RNA molecules) [180]. MiRNAs are transported between cells by exosomes and can either promote or inhibit tumor growth [181,182]. The activity of potassium ion channels is modulated, among other things, by hypoxia (see Section 2.3) and sialylation (see Section 3.2). At the same time, the level of sialyltransferases, enzymes responsible for attaching sialic acid to glycans, depends on hypoxia [183], and an increase in ST6Gal-I sialyltransferase levels correlates with an increase in HIF-1 factor expression [184]. Additionally, studies suggest that hypoxia is directly related to miRNAs that regulate mitochondrial function [185]. Therefore, we decided to look for miRNAs that, such as hypoxia, can affect the expression of both potassium channels and sialyltransferases. To do this, we searched three algorithmically different databases (TargetScan [75], MirTarBase [76,77] and MirDB [78]). The results, miR indicated for a given sialyltransferase and individual potassium channels by at least two databases, are shown in Table 2. Almost all selected (except miR-135, miR-1297, miR-4319, miR-8485) can be directly linked to hypoxia (Table 2). Furthermore, all of these miRNAs (except miR-4319 and miR-6838) are exosomal miRNAs (Table 2), so these can be extracellular elements that regulate sialylation and potassium channel levels and can also affect cellular pathways associated with hypoxia.

## 4. Discussion

This review highlights the relations between the potassium channels, glucose metabolism, and glycosylation in cancer cells, which can be useful in deciphering rarely considered aspects of the molecular pathophysiology of oncological diseases. Ion channels emerge as a promising group of proteins that can be considered drug targets in cancer treatment. Let us provide a few examples.

### 4.1. Pharmacological Modulation of Potassium Channels in Cancer

Several studies have investigated the potential use of potassium channel inhibitors as a therapeutic strategy for cancer. In fact, potassium channels are highly expressed in both the plasma membrane and the inner mitochondrial membrane of many forms of cancer [8]. These channels include the intermediate-conductance calcium-dependent potassium channel (K${}_{Ca}$3.1) expressed in the plasma membrane and inner mitochondrial membrane (mitoK${}_{Ca}$3.1). In particular, blocking mitoK${}_{Ca}$3.1 but not K${}_{Ca}$3.1 with the inhibitor TRAM-34 results in the in vitro death of tumor cells and reduces their metastatic spread in vivo [215]. The role of potassium channels located in the mitochondrial membrane has also been highlighted by the discovery of a new inhibitor of the K${}_{2P}$9.1 (TASK-3) channel. The mitochondriotropic version of this inhibitor (mitoIN-THPP) was seen to decrease the survival of breast cancer cells and kill melanoma cells, whereas IN-THPP was unable to do it, highlighting the importance of potassium channels located in the mitochondrial membrane as privileged pharmacological targets in the therapy of various forms of cancer [216]. Also in chronic lymphocytic leukemia (CLL) Kv1.3 and K${}_{Ca}$3.1 channels are highly expressed both in the plasma and mitochondrial membranes, and inhibiting mitoKv1.3 with PAPTP induces in vitro death of CLL cells, while inhibition of K${}_{Ca}$3.1 with TRAM-34 decreases their proliferation. The action of PAPTP was also exerted on CLL cells resistant to ibrutinib, and PAPTP also enhances the therapeutic action of Venetoclax by acting on mitoKv1.3. PAPTP also decreases CLL in vivo in animal models of CLL [217].

Other potassium channels, such as the voltage-gated potassium channels, are highly expressed in ductal pancreas adenocarcinoma (PDAC), and their blockade could be a useful therapeutic strategy to be added to conventional therapy [218]. Also in breast cancer, in particular the triple-negative type, it has been seen that the expression of potassium channels determines increased in vitro invasion, tumor growth in vivo, and metastases, so it is interesting to reposition already approved potassium channel-blocking drugs for the therapy of this type of particularly aggressive cancer [219,220]. In breast cancer, the expression of potassium channel subfamily K member 6 (K${}_{2P}$6.1) is also increased, which enhances the proliferation, invasion, and migratory capacity of cancer cells. Therefore, K${}_{2P}$6.1 can also be considered a target to be blocked for additional breast cancer therapies [221]. Another interesting finding is the cooperation between ion channels in promoting tumor growth. For example, the chloride intracellular channel 1 (CLIC1) cooperates with the potassium channel Kv10.2 (also called EAG2) in promoting the growth of medulloblastoma cells, and their simultaneous silencing synergistically suppresses tumor growth [222].

### 4.2. Is It Possible to Reprogram the Cancer Metabolism via Modulation of Potassium Channels?

Due to the characteristic switch in glucose metabolism from oxidative phosphorylation toward aerobic glycolysis, cancer can be considered a metabolic disease [37,38]. Moreover, the metabolic traits seem to depend on the stage of the oncological disease. Many metastases display metabolic differences in comparison with the primary tumors, as summarized in [223]. These changes enable the metastatic tumors to survive and grow in different environments. From this perspective, the idea of anti-glycolytic cancer therapy seems encouraging [224,225,226]. Nevertheless, the development of effective solutions of this kind is not an easy task due to the high energetic adaptability of cancer cells ensuring cell survival in terms of varying availability of energetic substrates, its metabolites, and enzymes for their conversions [16].

As described in this review, there are complex interconnections between potassium channels and metabolic reprogramming in cancer, which suggests that the K${}^{+}$ channels are one of the emerging molecular targets in anti-cancer therapies. Among the promising metabolic modulators that affect the functioning of potassium channels, one should mention dicholoroacetate (DCA), which already has been mentioned in Section 2.1. The DCA upregulates mitochondrial Kv1.5 channels by an NFAT1-dependent mechanism in cancer cells, which results in restoring OxPhos and triggering apoptosis [13]. Other interesting substances are mitochondria-targeted derivatives of PAP-1, well-known Kv1.3 channel inhibitor. It was shown that these derivatives, namely PAPTP and PCARBTP, lead to ROS-dependent apoptosis of cancer cells via inhibition of the mitoKv1.3 channel. What’s interesting is that the tested compounds did not affect healthy cells [10].

Considering the perspectives of $K^{+}$ channel modulation in the aim to suppress cancer, one possible approach is to exploit the existing relationships between $K^{+}$ levels and the activity of metabolic enzymes and transcription factors. In this context, the hypoxia-inducible factor 1-alpha can be considered a therapeutic target. It increases glycolytic enzymes’ expression (e.g., LDHA, HKs), and at the same time, its signaling correlates with the expression and activity of Kv10.1 and Kv11.1 channels [149,150,151,155]. Considering enzymes, one should mention the possible down-regulation of hexokinase II via the increased expression and activation of the Kv1.3 channel [27] or KCa3.1 [71]. Lowering HK2 levels can be beneficial due to the decrease in glycolytic activity of cancer cells [27] and reducing their metastasis [225]. The increased production of lactate from pyruvate, which is typical for cancer cells, is gathered by the elevated LDH enzymes (LDHA, LDHB). In particular, LDHA is considered a viable target for drug design and discovery [227,228] since it has a higher affinity for pyruvate, preferentially converting pyruvate to lactate, and NADH to NAD${}^{+}$ in anaerobic conditions. According to the studies [63], increased expression of the K${}_{2P}$3.1 channels can lead to down-regulation of LDHA.

As we summarized in Section 2.2, the mitochondrial potassium channels play important roles in the regulation of cell death processes. The molecular mechanism of their involvement is mainly associated with regulation of ROS production (like Kv1.3 channels [8,130,131]), but also they can modulate the oxidative phosphorylation (like K${}_{Ca}$3.1 channels [138]), cytoprotection (like mitoK${}_{ATP}$ and mitoBK${}_{Ca}$ channels [141,143]) and the cellular response to hypoxia (like Kv11.1 channels [149,150,151,155], K${}_{Ca}$ channels [229] including the BK${}_{Ca}$ ones [152], and the K${}_{2P}$ channels: K${}_{2P}$3.1 [153] and K${}_{2P}$9.1 [154]). This may inspire forthcoming research where the K${}^{+}$ channels in the mitochondrial membranes can be considered important targets for controlled induction of the pro-death processes in cancer cells.

As one can see, the metabolic changes in tumor cells are gathered with changes in K${}^{+}$ channels’ expressions and their transport properties across the plasma- and mitochondrial membranes. Nevertheless, we are still far from a complete understanding of the mechanistic picture of this phenomenon. For example, enhanced investigations on the mechanisms and functions of microRNAs in the ion channel regulation, which can affect cancer metabolism, are needed. They could allow for the discovery of additional prognostic biomarkers as well as support the effective development of novel therapeutic strategies. Here, we presented some new microRNA-oriented directions for further research. In Table 1, we presented the channel types whose expression can be affected by the microRNAs involved in the regulation of glucose metabolism in cancer cells. The presented nine types of microRNAs are the ones that are most probable to interfere with the $K^{+}$ channels, but they are definitely not the only ones that can exhibit mutual relations. Moreover, most of the selected microRNAs from Table 1 have been identified in exosomes. Our approach has a limitation because the cell-type match with the exosomal release of particular microRNAs still needs to be experimentally verified. Nevertheless, the sole possibility of the exosomal delivery of the indicated metabolism- and K${}^{+}$ channel-related microRNAs seems promising due to the anticipated profound effects on cell-cell communication that can facilitate tumor growth, invasion, metastasis, angiogenesis, and drug resistance.

### 4.3. The Therapeutic Potential of Controlling Potassium Channels Glycosylation

The activity and distribution of potassium channels in the cell affect proliferation, cell cycle, apoptosis, and tumor progression [6]. One of the factors that regulates the activity, distribution in the membrane, and stability of K${}^{+}$ channels is glycosylation [177]. In particular, the negatively charged sialic acid present in glycans affects the electrical excitability of cells [230]. Altered sialylation occurs in most cancer cells, and the presence of sialic acid-containing tumor antigens correlates with poor survival [231]. Inhibition of sialization reduces metastasis and increases cell sensitivity to chemotherapy and radiotherapy [232]. It has also been shown that an increase in ST6Gal-I sialyltransferase levels correlates with an increase in hypoxia-inducible factor HIF-1α and HIF-1α-regulated genes, including glucose transporter genes GLUT1 and GLUT3 and the glycolytic enzyme gene PDHK1 [184]. Selective inhibition of sialotransferases and other enzymes involved in the synthesis of sugar structures overexpressed in cancer cells can stop cancer growth and restore potassium channel activity to normal. Thus, it seems that enzymes involved in glycosylation, particularly sialotransferases, may be great targets for molecular therapy.

Another promising idea is to exploit oligosaccharides attached to potassium channels to deliver potassium channel-specific inhibitors/modulators directly to the channel environment. Such an approach can enable the delivery of appropriate channel modulators and their release it in situ via glycan-mediated pH-dependent click chemistry methodology, similarly to the procedure described in [233].

The main regulatory elements of glycome in the cell are miRNAs [234]. The miRNAs are an interesting potential target for channel therapy in that there are miRNAs correlated with hypoxia that can simultaneously regulate the expression of enzymes involved in oligosaccharide synthesis (Table 2). Moreover, some of these miRNAs also regulate glycolysis (Table 1). Introduced into cells, miRNAs could therefore comprehensively affect both the expression of potassium channels and the metabolism of cancer cells. It is worth noting that most of the miRNAs we identified as miRNAs that could simultaneously regulate the expression of either of the sialotransferases and either of the potassium channels are exosomal miRNAs (Table 2). Exosomes are microvesicles that are part of intercellular communication present in physiological fluids [235]. There is a cell-type-specific mechanism for packaging miRNAs into exosomes [236]. Furthermore, the content of exosomes secreted by normal cells differs from the content of exosomes secreted by cancer cells,; hence, exosomal miRNAs may serve as a readily available biomarker of cancer [237].

### 4.4. Challenges and Perspectives

Finding new drugs that reprogram cancer metabolism and act on plasma membrane- and mitochondrial channels seems like a viable but challenging idea. Among the most problematic issues, one should mention that most of the relations between potassium channels and the biomolecules involved in the regulation of glucose metabolism are indirect. Thus, the response to channel modulation can be complex and needs an in-depth experimental inspection. Moreover, most types of cancer exhibit metabolic plasticity. Therefore, even if channel activation/blocking could be beneficial from the point of view of its interaction with a particular enzyme/transcription factor, or other mediating molecule (e.g., glucose intake and aerobic glycolysis) cancer cells are able to adapt to unfavorable environmental or cellular conditions by reprogramming their metabolism to survive and sustain proliferation.

Additional challenges in channel-oriented drug design refer to their specificity and possible undesired cytotoxicity outside the tumor. A possible problem with potassium channel blockers/activators is their poor selectivity due to structural similarities between the various channel types. For example, Kv10.1, highly expressed in 70% of human tumors but not in healthy tissue outside the brain, is very similar to Kv11.1 (also called hERG). It is therefore necessary to look for drugs that act with higher selectivity by studying the differences between these channels [238]. Nevertheless, different types of cancer may involve different mechanisms of $K^{+}$ channel activation depending on the origin of tumor. There are several very interesting tissues associated with ion channel specificity in the cancer context. First of all, the environment of cancer, including hypoxia, leads to the changeability of the activation of different channel types. Moreover, an extremely important and not sufficiently studied aspect is the impact of potassium channel mutations in various types of cancer [7]. The expression level of the specific potassium channels is inversely proportional to the level of tumor malignancy in glioma, lymphoma, or colorectal cancer [239,240,241,242].

In this review, we paid much attention to mitochondrial potassium channels. Their druggability is a complex issue [243]. Due to the structural and functional adaptations of the mitoK channels to operate in terms of relatively high membrane potential, high Ca${}^{2+}$ concentrations, and alkaline pH in the mitochondrial matrix, they display unique regulatory properties. Nevertheless, the specificity of the openers and inhibitors to mitochondrial channel isoforms, the possibility of drug delivery to the mitochondrion and their accumulation within the matrix, as well as precluding drug interactions with the electron transport chain, are challenging problems for further investigations [243].

Another challenge for channel-oriented anti-cancer therapy is to recognize and control the possible non-channel off-targets. Sometimes, the channel modulators act in a pleiotropic manner. For example, the CGS7184, a mitoBK${}_{Ca}$ channel opener, induces cell death in neuronal cells. This effect is, however, related to the deregulation of calcium homeostasis by CGS7184 via, at least partly, activation of calpain proteases [244]. Analogously, the 3-chloro-4-methoxyphenyl derivative of bromotyramine purpurealidin E [245] being an effective Kv10.1 inhibitor shows dose-dependent cytotoxic and proapoptotic effects. Nevertheless, these effects are detectable both on Kv10.1 expressing- and non-expressing cell lines. Thus, they involve independent mediating biomolecules. Moreover, these cytotoxic and proapoptotic effects were also not restricted to cancer cell lines, which hinders the usage of this Kv10.1 inhibitor in cancer treatment.

It is also important to underline the role of pharmacological modulation of potassium channels to combat the undesirable effects of some drugs used in the treatment of cancer. For example, gemcitabine, commonly used in the treatment of pancreatic cancer and non-small cell lung cancer, may cause cardiotoxicity as it reduces the expression of Kv11.1 (human rapid delayed rectifier potassium channel, hERG). Therefore, by using hERG as a pharmacological target to increase its expression, it can be thought to reduce the cardiotoxicity of gemcitabine [246]. Another example is the drug oxaliplatin, which produces neurotoxicity as an adverse effect. In experimental models, riluzole prevents neurological deficits caused by oxaliplatin due to its action on the potassium channel K${}_{2P}$2.1 (TREK-1) [247].

A possible solution to some of the aforementioned problems is drug repositioning [248]. The drug repurposing technique, which frequently uses artificial intelligence, has also been used to identify drugs blocking the Kv11.1 K${}^{+}$ channels that have a role in the proliferation, survival, secretion of angiogenic factors, invasiveness, and metastasis of cells of various tumor types (epithelial, neuronal, leukemic and connective tissue). By using this technique, 26 already approved drugs have been identified, including, for example, bromocriptine, darglitazone, and troglitazone, that have a tumor inhibition effect, although further studies are needed to understand their mechanism [249]. Two other drugs, such as loperamide and amitriptyline, have been identified through a thallium influx-based assay and have been shown to block potassium channels Kv10.1 that induce the proliferation of different tumor cell types [250].

Another possible solution to the existing problems in channel-oriented anti-cancer drug design is to target only the cancer-specific glycosylated channel variants. In such an approach, the particular glycans (e.g., the sialylated N-glycans) can serve as anchoring points for channel modulator-directed chemistries [233]. To enable the utilization of this methodology, several issues should be explored. For example, the appropriate channels’ glycan attachment sites should be recognized to ensure the saccharide-specificity of the glycoproteomic method.

Considering glycosylated forms of ion channels, once again microRNAs deserve a particular scientific interest. According to our literature and database analysis (see Table 2, based on the analysis of TargetScan [75], MirTarBase [76,77], and MirDB [78]), some microRNAs are anticipated to affect both the expression of sialyltransferases responsible for forming sialylated protein glycans in terms of hypoxia and the potassium channels at the transcriptional level. This relation is particularly interesting in the case of the Kv4.3 channels. The glycolysation of their structure is important for cancer biology, as described in Section 3.2. In turn, the analysis of microRNA repositories allowed us to find the possible enzyme involved in binding saccharides to their structures (i.e., alpha-2,8-Sialyltransferase III, ST8SIA3), as well as the regulatory microRNAs (miR-148, miR-152), Table 2. Both miRs are related to hypoxia [194,195] and may be present in cancer-derived exosomes [189,196], which needs, however, experimental validation.

## 5. Conclusions

In this work, we discussed novel metabolism- and glycome-related aspects of tumor biology, with particular attention paid to the role of the expression and activity of potassium channels. The recognized complex interconnections between the channels’ functioning and metabolic alterations allow us to consider cancer a metabolic disease and a channelopathy. Therefore, the research of detailed mechanisms responsible for the involvement of plasma membrane- and mitochondrial channels in cancer energetics as well as the potential saccharide-mediated drug delivery to cancer-specific ion channel glycans are promising fields to explore in further biochemical and pharmacological investigations. Among the other rising directions for further research, we would like to particularly distinguish the microRNAs as viable cancer biomarkers and potential effective drug targets since they regulate both aspects of cancer biology—the metabolic switch and altered glycosylation, which have been discussed in this work.

## Figures and Tables

**Table 1:** The microRNAs anticipated as the common regulators of the expression of particular potassium channels and molecular targets responsible for reprogramming glucose metabolism in cancer. The involvement of microRNAs in glycolytic pathways in particular cancer models was confirmed experimentally. The arrows symbolize the observed changes in microRNA expression: ↓ downregulation, ↑ upregulation. The regulation of the expression of ion channels by a given microRNA was indicated according to the predictions from at least two of three independent repositories TargetScan [75], MirTarBase [76,77] and MirDB [78]. For each microRNA, we present also the references which determine whether a given microRNA could be present in exosomes ([miRNA–Exosomes]). Table is partly adapted from [74] with permission from Elsevier (2023).

Potassium Channels	microRNA	Target	Cancer (Model)	Expression	References	References [miRNA–Exosomes]
Kir2.1, K${}_{2P}$4.1	miR-9-5p	HK2	Colorectal cancer (human)	↑	[79]	[80,81]
Kir2.2	miR-603	HK2	Ovarian cancer (in vitro, in vivo, human)	↑	[82]	–
			Ovarian cancer (human)	↓		
Kir3.1	miR-361-5p	Sp1/PKM2	Bladder cancer (in vitro)	↓	[83]	[84]
Kv12.3, Kv9.3	miR-125a-5p	CD147	Thyroid cancer (in vitro)	↓	[85]	[86,87,88,89]
Kv1.1, Kv12.3, Kv9.3	miR-125b-5p	HK2	Laryngeal squamous (in vitro, human)	↓	[90]	[91,92]
Kv1.2	miR-137	NOX4	Prostate cancer (in vitro)	↑	[93]	[94,95]
		GLO1	Melanoma (in vitro)	↓	[96]	
Kv7.3	miR-449a	LDHA	Lung cancer (in vitro)	↓	[97]	[98,99]
Kv7.5	miR-139-5p	HK1, PFKFB3	Liver cancer (in vitro, in vivo)	↓	[100]	[101,102]
		PRKAA1	Gastric cancer (in vitro)	↓	[103]	
KCa3.1	miR-15b-5p	PDK4	Osteosarcoma (in vitro)	↓	[104]	[105,106]

**Table 2:** The microRNAs anticipated as the common regulators of the expression of particular sialyltransferases and potassium channels. The microRNAs were indicated by at least two of three independent repositories (TargetScan [75], MirTarBase [76,77] and MirDB [78]). For each microRNA we present the references which describe its association with hypoxia ([miRNA–Hypoxia]) and/or state that it is an exosomal microRNA ([miRNA–Hypoxia]).

microRNA	Sialyltransferase	Potassium Channel	References [miRNA–Hypoxia]	References [miRNA–Exosomes]
miR-15	ST8SIA3	K${}_{Ca}$3.1	Xue et al. (2015) [186]	Luo et al. (2022) [187]
miR-16	ST8SIA3	K${}_{Ca}$3.1	Xue et al. (2015) [186]	Luo et al. (2022) [187]
miR-26	ST6GAL2	Kv11.3	Li et al. (2021) [188]	Chettimada et al. (2020) [189]
		Kv7.4		
		K${}_{2P}$1.1		
miR-122	ST6GALNAC4	Kir7.1	Xu et al. (2022) [190]	Xu et al. (2022) [190]
miR-125	ST6GAL1	Kv1.1	Li et al. (2018) [191]	Kot et al. (2023) [192]
		Kv9.3		
		Kv12.3		
	ST6GALNAC6	Kv1.1		
		Kv12.3		
		Kv9.3		
miR-135	ST6GAL2	Kv4.1	—	Parikh et al. (2021) [193]
		Kir3.2		
	ST8SIA3	Kv4.1		
		Kir3.1		
miR-148	ST8SIA3	Kv4.3	Behara et al. (2023) [194]	Chettimada et al (2020) [189]
		Kir3.1		
miR-152	ST8SIA3	Kv4.3	Zhao et al. (2021) [195]	Li et al. (2022) [196]
miR-190	ST6GAL2	Kv7.5	Blissenbach et al. (2018) [197]	Sotillo et al. (2020) [198]
miR-195	ST8SIA3	K${}_{Ca}$3.1	Lin et al. (2021) [199]	Cheng et al. (2022) [200]
miR-218	ST8SIA5	Kv4.2	Xu et al. (2022) [201]	Cheng et al. (2022) [200]
		K${}_{2P}$15.1		
miR-365	ST6GAL2	Kir3.1	Zhou et al. (2018) [202]	Coon et al. (2020) [203]
		Kv11.1		
		Kv7.1		
miR-377	ST6GALNAC5	Kv1.4	Cui et al. (2019) [204]	Wang et al. (2022) [205]
miR-424	ST8SIA3	K${}_{Ca}$3.1	Tsai et al. (2018) [206]	Wang et al. (2022) [207]
miR-497	ST8SIA3	K${}_{Ca}$3.1	Ye et al. (2022) [208]	Abdelrahma et al. (2022) [209]
miR-670	ST8SIA3	Kv9.1	–	Lin et al. (2022) [210]
miR-1297	ST6GAL2	Kv11.3	–	Luo et al. (2021) [211]
		Kv7.4		
		K${}_{2P}$1.1		
miR-4319	ST6GAL1	Kv9.3	–	–
		Kv12.3		
	ST6GALNAC6	Kv12.3	–	–
		Kv9.3		
miR-4465	ST6GAL2	Kv7.4	Cao et al. (2021) [212]	Cao et al. (2021) [212]
		K${}_{2P}$1.1		
miR-6838	ST8SIA3	K${}_{Ca}$3.1	Zhang et al. (2022) [213]	–
miR-8485	ST8SIA4	Kir2.2	–	Li et al. (2020) [214]
		Kir3.1		
		Kir4.1		
		Kv1.4		
		Kv2.1		
		Kv3.4		
		K${}_{2P}$5.1		
		K${}_{2P}$10.1		

## Data Availability

Not applicable.

## Abbreviations

The following abbreviations are used in this manuscript:

Acetyl-CoA	acetyl coenzyme A
ADP	adenosine diphosphate
ATP	adenosine triphosphate
BK${}_{Ca}$	big conductance Ca${}^{2+}$-activated potassium channel
CLIC1	chloride intracellular channel 1
DCA	dichloroacetate
Eno	enolase
ETC	electron transport chain
FADH	reduced form of flavin adenine dinucleotide
G6P	glucose-6-phosphate
GDH	glutamate dehydrogenase
GLS	glutaminase
GLT-1	glutamate transporter-1
GSH	glutathione
hERG	human rapid delayed rectifier potassium channel
HIF-1	hypoxia-inducible-factor-1
HK1 (HKI)	hexokinase-1
HK2 (HKII)	hexokinase-2
HO-1	heme oxygenase-1
Hsp90	Heat Shock Protein 90
IKCa	intermediate calcium activated potassium channel
$K_{2P}$	two-pore domain potassium channel
$K_{Ir}$	inward rectifier potassium channel
K${}_{V}$	voltage-gated potassium channel
LDHA	lactate dehydrogenase A
LCSC	liver cancer stem cells
MCT4	monocarboxylate transporter-4
mitoK${}_{ATP}$	mitochondrial ATP-sensitive potassium channel
mitoBK	mitochondrial big potassium channel
mitoBK${}_{Ca^{2+}}$	Ca${}^{2+}$-activated mitochondrial big potassium channel
mitoK${}_{ATP}$	ATP-sensitive mitochondrial potassium channel
mitoSK${}_{Ca}$	Ca${}^{2+}$-activated mitochondrial small-conductance potassium channel
mitoSLO2	mitochondrial sodium-activated potassium channel
mitoTASK-3	mitochondrial TWIK-related acid-sensitive potassium channel
MPC	mitochondrial pyruvate carrier
MPTP	mitochondrial permeability transition pore
NADH	reduced form of Nicotinamide adenine dinucleotide
NOX4	NADPH oxidase 4
O-GLCNAc	O-linked-N-Acetyl-D-glucosamine
O-GlcNAcylation	O-linked-N-acetylglucosaminylation
OxPhos	oxidative phosphorylation
PAH	pulmonary artery hypertension
PDAC	ductal pancreas adenocarcinoma
PDH	pyruvate dehydrogenase
PDK1	pyruvate dehydrogenase kinase-1
PDK4	pyruvate dehydrogenase kinase-4
PEP	phosphoenolopyruvate
PFK1	6-phosphofructokinase-1
PFKFB3	6-phosphofructo-2-kinase/fructose-2,6-biphosphatase 3
PGI	phosphoglucose isomerase
PGK	phoglycerate kinase
PGM	phosphoglycerate mutase
PK	pyruvate kinase
PKM2	pyruvate kinase isoenzyme type M2
PRKAA1	AMP-activated, alpha 1 catalytic subunit
ROS	reactive oxygen species
TCA	tricarboxylic acid (cycle)
SK	small conductance calcium-activated potassium channel
Sp1	transcription factor specificity protein 1
TME	tumor microenvironment
UDP-GlcNAc	UDP-N-acetylglucosamine
VDAC	voltage-dependent anion channel
VEGF	vascular endothelial growth factor
